# Knowledge, opinions, and practices related to oral cancer prevention and oral mucosal examination among dentists in Moldova, Belarus and Armenia: a multi-country cross-sectional study

**DOI:** 10.1186/s12903-021-02011-2

**Published:** 2021-12-18

**Authors:** Olga Golburean, Maria Helene Hagen, Diana Uncuta, Marcela Tighineanu, Gayane Manrikyan, Izabella Vardanian, Christoffer Andresen, Bhavdeep Singh, Tatiana Porosencova, Irina Ivasiuc, Olga Cheptanaru, Marina Markaryan, Natalia Shakavets, Dipak Sapkota, Tine Merete Søland, Daniela-Elena Costea, Ferda Özkaya

**Affiliations:** 1 Centre for International Health, Faculty of Medicine, University of Bergen, Bergen, Norway; 2 Department of Clinical Dentistry, Faculty of Medicine, University of Bergen, Bergen, Norway; 3 Department of Stomatological Propedeutics “Pavel Godoroja”, Faculty of Stomatology, State University of Medicine and Pharmacy “Nicolae Testemiţanu”, Chisinau, Moldova; 4 Department of Therapeutic Stomatology, Faculty of Stomatology, Yerevan State Medical University, Yerevan, Armenia; 5 Institute of Oral Biology, Faculty of Dentistry, University of Oslo, Oslo, Norway; 6 Department of Pediatric Dentistry, Faculty of Dentistry, Belarusian State Medical University, Minsk, Belarus; 7 Department of Pathology, Rikshospitalet, Oslo University Hospital, Oslo, Norway; 8 Department of Clinical Medicine, Center of Cancer Biomarkers CCBIO, Faculty of Medicine, University of Bergen, Bergen, Norway; 9 Department of Pathology, Haukeland University Hospital, Bergen, Norway

**Keywords:** Oral cancer, Prevention, Knowledge, Opinion, Practice, Dentist, Moldova, Belarus, Armenia

## Abstract

**Introduction:**

Moldova, Belarus, and Armenia are post-Soviet countries with a high rate of heavy smokers and a relatively high age-standardized incidence of oral cancer. However, to our knowledge, there is lack of available information on dentists’ knowledge on prevention of oral cancer in the countries in question. Accordingly, this study aimed to assess the knowledge, opinions, and practices related to oral cancer prevention and oral mucosal examination among dentists in Moldova, Belarus, and Armenia.

**Methods:**

This was a multi-country, cross-sectional study based on a self-administered questionnaire. A structured questionnaire was distributed to 3534 dentists (797 in Chisinau, Moldova, 1349 in Minsk, Belarus, and 1388 in Yerevan, Armenia). Dentists' knowledge about risk factors for oral cancer development and its clinical picture, current practices and opinions with regard to oral mucosal screening and oral cancer prevention, and their consistency to perform oral mucosal examination were assessed. A knowledge score ranging from 0 to 14 points was generated based on each dentist’s answer to the questionnaire.

**Results:**

A total of 1316 dentists responded, achieving an overall response rate of 37.2% (34.5% in Moldova; 52.3% in Belarus; 24.2% in Armenia). Most dentists in the three countries correctly identified tobacco (83.8–98.2%) and prior oral cancer lesions (84.0–96.3%) as risk factors for oral cancer. Most dentists correctly identified leukoplakia as a lesion with malignant potential (68.7% in Moldova; 88.5% in Belarus; 69.9% in Armenia), while erythroplakia was identified by much fewer in all three countries. Less than 52% of dentists identified the tongue, rim of tongue, and floor of mouth as the most common sites for oral cancer. The mean knowledge score for all countries combined was 7.5 ± 2.7. The most commonly reported barriers to perform oral mucosal examination were lack of training, knowledge, and experience.

**Conclusions:**

This study highlights the need for improved oral cancer-related education and training on oral mucosal examination for dentists in Moldova, Belarus, and Armenia. Such skills are essential to enhance oral cancer prevention and to improve the prognostic outcome by early detection.

**Supplementary Information:**

The online version contains supplementary material available at 10.1186/s12903-021-02011-2.

## Introduction

Oral squamous cell carcinoma (OSCC), a malignancy arising from the surface epithelium of oral cavity, accounts for more than 90% of all oral cancers [1]. As the estimated 5-year survival rate for OSCC significantly decreases from approximately 85% if detected at early stages (I and II) to 40% if detected at advanced stages (III and IV) [2], its early detection is crucial. It is consequently essential that oral health practitioners understand the importance of conducting a thorough oral mucosal examination for malignant and potentially malignant lesions as part of their routine clinical assessments, including the examination of high-risk areas such as the rim of the tongue and the floor of the mouth [3, 4]. Having a good knowledge of the risk factors of OSCC such as tobacco use, excessive alcohol consumption, exposure to ultraviolet radiation, a past positive OSCC history, advanced age, and a poor diet/nutrition may also increase the awareness of the dentists and thereby detection of OSCC at an early stage [5–9].

Several studies from Europe, North America, Australia, and the Middle East identified gaps in dentists’ knowledge related to the risk factors, clinical presentation, and diagnostic procedures of oral cancer, as well as to the guidelines and protocols for referral of patients with lesions with malignant potential [3, 10–20]. Thus, there is a worldwide need for improved knowledge and education of oral health practitioners on early diagnosis and referral of OSCC [9, 21, 22]. This will have an overall positive impact on improving patients’ quality of life, decreasing treatment costs, and reducing morbidity and mortality rates.

Moldova, Belarus, and Armenia are all post-Soviet nations with a high rate of heavy smokers in their populations and a relatively high age-standardized incidence rate of OSCC [23]. For Moldova, the prevalence of adult smokers was 25.3% in 2013 (men: 43.6%; women: 5.6%) [24], and oral cancer accounted for 1.9% of all new cancer cases and for 1.9% of all cancer-related deaths in 2018 [25]. Interestingly, there has been a 22% increase in oral cancer-related mortality in women in Moldova between 1990–1994 and 2005–2007 [23]. In Belarus, the prevalence of adult smokers in 2013 was 25.9% (men: 48.6%; women: 9.7%) [26], and even more alarming is the finding of a 61% increase in oral cancer-related mortality in women between 2000–2003 and 2007 [23]. In 2018, oral cancer accounted for 1.6% of all new cancer cases and for 1.7% of all cancer-related deaths in Belarus [27]. For Armenia, where the prevalence of adult smokers was 25.4% in 2012 (men: 50.9%; women: 3.2%) [28], oral cancer accounted for 0.50% of all new cancer cases and for 0.41% of all cancer-related deaths in 2018 [29]. Moldova and Belarus also have very high levels of alcohol consumption, with a per capita alcohol consumption of 15.2 L for Moldova and 11.2 L for Belarus in 2016 compared to 5.5 L for Armenia and 9.8 L for the overall European region [30].

To our knowledge, there is lack of available information on dentists’ knowledge on oral cancer in the countries in question. Accordingly, the aim of this study was to assess the knowledge, opinions, and practices related to oral cancer prevention and oral mucosal examination among dentists in the capital cities of Moldova (Chisinau), Belarus (Minsk), and Armenia (Yerevan).

## Materials and methods

### Study design

This multi-country cross-sectional study was conducted using a structured, self-administered questionnaire that was distributed to all dental clinics in Chisinau, Minsk and Yerevan. The study received ethical approval from the Norwegian Centre for Research Data (Project No. 471282 and 57451), and national ethical committees in Moldova, Belarus, and Armenia. Participation in this study was voluntary, and written informed consent was obtained from all participating dentists. The data collection period was from June 2018 to September 2019.

All actively practicing dentists in Minsk, Chisinau and Yerevan were invited to participate to the study. Prior to data collection, a list of 216 dental clinics in Minsk, 233 in Chisinau, and 130 in Yerevan was obtained from local coordinators, who were highly knowledgeable about local regulations and practices in dentistry. All dental clinics on the list were visited by the responsible investigator(s) who explained the purpose of the study and personally distributed the questionnaire to the consenting dentists. The responsible investigator(s) returned to each dental clinic after 1–3 days from the initial visit to collect the completed questionnaires.

### Questionnaire

The questionnaire used in the present study was developed based on previous studies conducted elsewhere [31–34]. The questionnaire was first prepared in English, and then translated into the local languages in the respective countries (Romanian, Russian, and Armenian), following a standardized forward–backward procedure. A pilot version of the questionnaire was tested on 10 dentists from each of the three countries during December 2017. Based on the pilot survey results and feedback from academicians at the collaborative institutions, necessary adjustments of the questionnaire were made.

The questionnaire, which was self-explanatory and closed-ended, consisted of 70 items, divided into six different parts: (1) personal data, (2) oral hygiene, dietary behavior, and utilization of dental services, (3) competency and orientation in preventive care, (4) preventive knowledge, (5) preventive practice for patients and (6) oral mucosal screening and oral cancer prevention. The later part on oral mucosal screening and oral cancer prevention was used in the analysis of the present study (Additional file 1). Clinical practices and opinions related to oral mucosal examination and oral cancer prevention, dentists’ barriers to oral mucosal examination, knowledge of oral cancer risk factors and diagnostic procedures, and oral cancer information sources were assessed. Response formats included a 5-point Likert scale, several correct answers in case of multiple-choice questions, and a ‘yes’, ‘no’, and ‘do not remember’ format. Questions (22–30) were used to assess dentists` level of knowledge regarding oral cancer. For each correct answer on the questions (22–30), a score of “1” was given. Dentists` level of knowledge was constructed based on the total number of points accumulated (ranging from 0 to 14). The present study used the mean score as cut-off point. The mean ± SD knowledge score for all countries combined was 7.5 ± 2.7 (range, 0–14). Knowledge score was dichotomized into 0 = lower score of knowledge (0–7), and 1 = higher score of knowledge (8–14).

### Data analysis

Data were analyzed separately for each country. All analyses were performed using IBM SPSS Statistics version 25.0 (IBM Corporation, Armonk, NY, USA). Descriptive statistics were reported using means and standard deviations (SD) for continuous variables and frequency with percentages for categorical variables. Chi-square tests were employed to assess bivariate relationships. Multivariable logistic regression analysis (adjusted odds ratio and 95% confidence intervals) was used to detect associations between the dentists’ knowledge score and their sociodemographic and work characteristics. The results that were statistically significant in the unadjusted analysis were included in the model. The analysis for the associations was done with merged data (from all countries). The level of statistical significance was defined as p < 0.05.

## Results

### Characteristics of the study participants

Of the 3534 dentists (797 in Moldova, 1349 in Belarus, and 1388 in Armenia) invited to participate, 1316 responded, giving an overall response rate of 37.2%. Out of the three countries, Belarus had the highest response rate at 52.3% (705/1349) compared to 34.5% (275/797) for Moldova and 24.2% (336/1388) for Armenia.

The sociodemographic and work characteristics of the study participants are presented in Table 1. Participants’ characteristics were overall comparable across the three countries, with some minor differences. For instance, in Belarus, the study population was predominantly male (79.1%), whereas a balanced number of women and men was found for Moldova and Armenia. Moreover, most dentists in Moldova (62.2%) and in Armenia (78.9%) were working exclusively in private clinics, while in Belarus, the majority were working in the public sector (60.9%). Only 8.0% of dentists in Moldova answered that they worked solo compared to 29.5% in Belarus and 42.3% in Armenia. More than half of dentists in Moldova (57.1%) and Armenia (61.6%) were general dental practitioners, whereas in Belarus, more than one-third (34.3%) were identified as restorative dentists or endodontists.

**Table 1:** Sociodemographic and work characteristics of study participants by country

	Moldova (N = 275)	Belarus (N = 705)	Armenia (N = 336)
Mean ± SD age (years)	40.3 ± 11.9	40.6 ± 11.2	38.2 ± 11.3
Age group (years)			
20–39	139 (50.5)	331 (47.0)	191 (56.8)
≥ 40	121 (44.0)	363 (51.5)	125 (37.2)
Gender			
Male	141 (51.3)	558 (79.1)	169 (50.3)
Female	134 (48.7)	143 (20.3)	167 (49.7)
Years of practice			
< 5	54 (19.6)	93 (13.2)	65 (19.3)
5–15	87 (31.6)	233 (33.0)	120 (35.7)
> 15	110 (40.0)	358 (50.8)	106 (31.5)
Work sector			
Private	171 (62.2)	162 (23.0)	265 (78.9)
Public/university	74 (26.9)	429 (60.9)	38 (11.3)
Both	28 (10.2)	113 (16.0)	23 (6.8)
Practice setting			
Solo	22 (8.0)	208 (29.5)	142 (42.3)
Non-solo	252 (91.6)	456 (64.7)	184 (54.8)
Specialty			
General dentistry	157 (57.1)	182 (25.8)	207 (61.6)
Restorative/endodontics	19 (6.9)	242 (34.3)	12 (3.6)
Prosthodontics	16 (5.8)	84 (11.9)	30 (8.9)
Oral surgery	14 (5.1)	34 (4.8)	19 (5.7)
Pediatrics/orthodontics	11 (4.0)	135 (19.1)	23 (6.8)
Periodontics	4 (1.5)	15 (2.1)	4 (1.2)
Oral pathology	3 (1.1)	11 (1.6)	3 (0.9)
Main patient group			
Children (0–18)	7 (2.5)	130 (18.4)	11 (3.3)
Adults	118 (42.9)	370 (52.5)	139 (41.4)
Mixed	148 (53.8)	195 (27.7)	174 (51.8)

Data are expressed as n (%), unless otherwise indicated. Percentages are calculated as n/N

Some figures are subject to missing data; values may not add up to total sample

*SD* standard deviation

### Knowledge of the clinical diagnosis of and risk factors for oral cancer

**Fig. 1:**
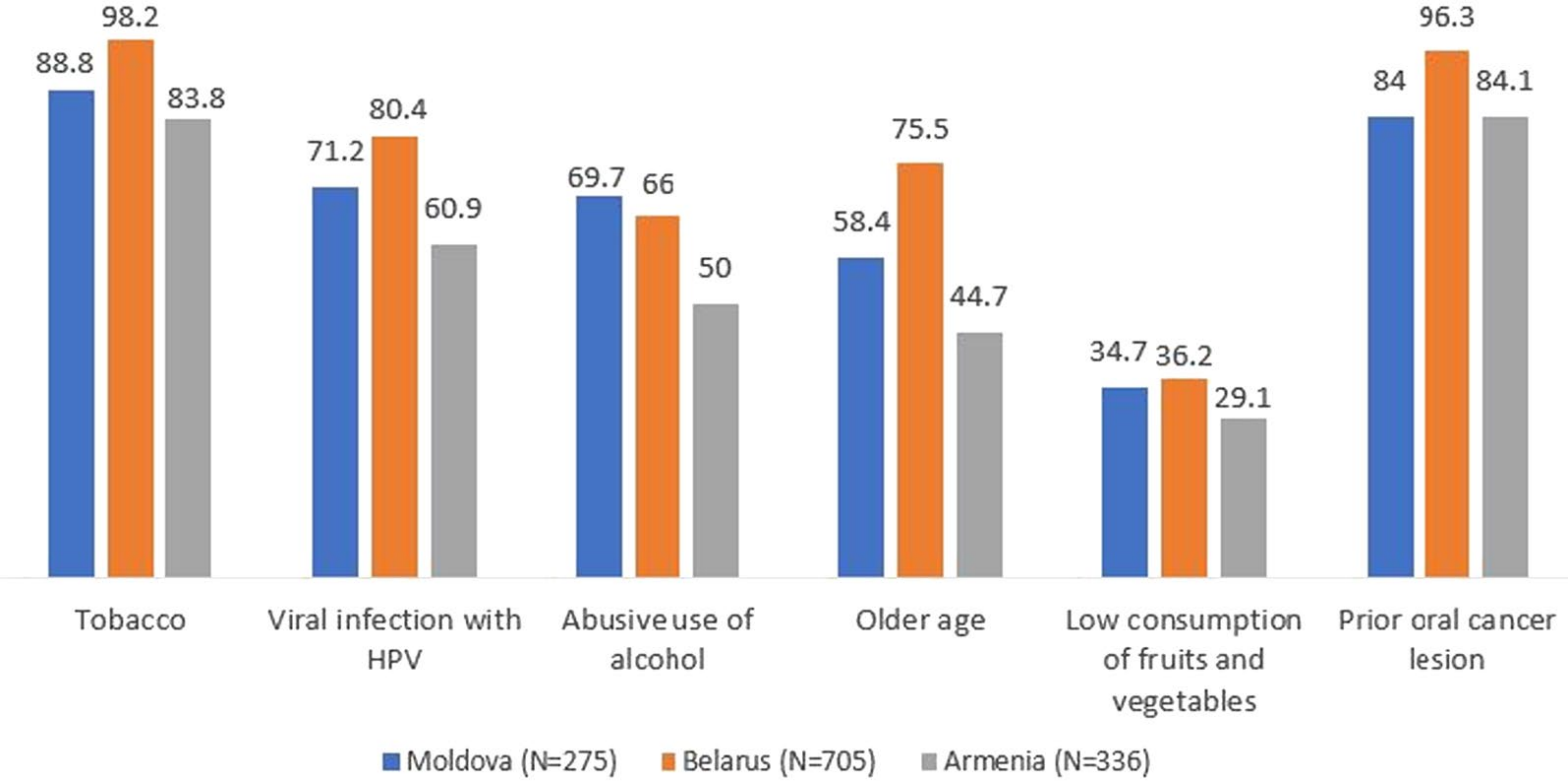
Percentage of dentists that correctly identified the risk factors for oral cancer

As shown in Fig. 1, most participants correctly identified tobacco (83.8–98.2%) and prior oral cancer lesions (84.0–96.3%), as risk factors for oral cancer. A lower percentage of dentists (50–69.7%) correctly identified abusive use of alcohol as a risk factor (Fig. 1).

When asked about the most common sites for oral cancer (Table 2), the tongue was correctly listed by 40.0% of dentists in Moldova, 51.8% in Belarus and 43.5% in Armenia. The floor of the mouth was also correctly identified by 31.6% of dentists in Moldova, 43.3% in Belarus, and 22.9% in Armenia. Less than 30% of the dentists were able to identify the rim of the tongue as one of the most common sites for oral cancer. Leukoplakia was considered the most common oral potentially malignant lesion in all three countries, listed by 68.7%, 88.5% and 69.9% of the dentists in Moldova, Belarus and Armenia, respectively. Erythroplakia was listed as the second lesion most likely to be oral potentially malignant lesion in Moldova and Belarus, while in Armenia, aphthous ulceration was listed as the second one. The most commonly listed clinical properties of early cancer lesions were small, painless and indurated ulcerations (33.9–63.4%) followed by small, painless white area (29.2–34.5%).

**Table 2:** Dentists' knowledge about the clinical diagnosis of oral cancer

	Moldova (N = 275)	Belarus (N = 705)	Armenia (N = 336)
Most common sites for oral cancer			
All sites equally	93 (33.8)	156 (22.1)	90 (26.8)
* FloFor of the mouth*	87 (31.6)	305 (43.3)	77 (22.9)
Buccal/lip mucosa	107 (38.9)	268 (38.0)	134 (39.9)
Hard palate	55 (20.0)	79 (11.2)	92 (27.4)
Soft palate	51 (18.5)	73 (10.4)	47 (14.0)
Retromolar region/palatopharyngeal arches	48 (17.5)	120 (17.0)	32 (9.5)
* Tongue*	110 (40.0)	365 (51.8)	146 (43.5)
* Rim of tongue*	79 (28.7)	167 (23.7)	82 (24.4)
Do not know	34 (12.4)	49 (7.0)	29 (8.6)
Oral potentially malignant disorders			
Morbus Crohn	27 (9.8)	79 (11.2)	14 (4.2)
* Erythroplakia*	84 (30.5)	346 (49.1)	69 (20.5)
Blue nevus	45 (16.4)	167 (23.7)	40 (11.9)
* Leukoplakia*	189 (68.7)	624 (88.5)	235 (69.9)
Aphtha	55 (20.0)	63 (8.9)	135 (40.2)
Do not know	34 (12.4)	47 (6.7)	43 (12.8)
Clinical properties of an early cancer lesion			
* Small, painless white area*	94 (34.2)	243 (34.5)	98 (29.2)
* Small, painless red area*	65 (23.6)	125 (17.7)	70 (20.8)
* Small, painless, indurated ulceration*	155 (56.4)	447 (63.4)	114 (33.9)
Small, painful, indurated ulceration	49 (17.8)	189 (26.8)	86 (25.6)
Do not know	39 (14.2)	19 (2.7)	60 (17.9)

Data are expressed as n (%). Percentages are calculated as n/N

The underlined and italicized responses represent the correct answers

The mean ± SD score for knowledge of the risk factors and clinical diagnosis of oral cancer was 7.3 ± 2.9, 8.2 ± 2.2, and 6.2 ± 2.8 in Moldova, Belarus and Armenia, respectively. The mean ± SD knowledge score for all countries combined was 7.5 ± 2.7 (range, 0–14). Multivariable logistic regression analyses revealed that there was a significant association (p < 0.05) between dentists’ knowledge score and country as well as gender; female dentists and dentists from Belarus were significantly more likely to have a higher knowledge of the risk factors and clinical diagnosis of oral cancer (Table 3).

**Table 3:** Proportion of dentists with a knowledge score ≥ 8, and multivariate association between dentists’ characteristics and the knowledge score

	%	Adjusted OR (95% CI)
Country		
Moldova	51.2	1
Belarus	64.7	1.5 (1.1–2.1)*
Armenia	31.5*	0.4 (0.3–0.6)*
Gender		
Female	59.1	1
Male	43.8*	0.7 (0.5–0.9)*
Work sector		
Private	46.4	1
Public/university	60.8	1.0 (0.8–1.3)
Both	58.5*	1.1 (0.7–1.6)

Chi-square test; *CI* confidence interval, *OR* odds ratio

^*^p < 005

### Practices related to oral cancer prevention and early detection

Regarding dentists’ practices related to oral cancer prevention and early oral cancer detection, the majority reported that they examine all new patients (88.0% in Moldova, 94.6% in Belarus, and 72.0% in Armenia) as well as all recall patients (87.0% in Moldova, 84.2% in Belarus, and 67.3% in Armenia) for oral mucosal lesions. Moreover, most dentists (81.5% in Moldova, 77.3% in Belarus, and 87.1% in Armenia) reported that when taking a patient’s medical history, they ask about current/previous use of tobacco. By contrast, only 36.5% of dentists in Belarus reported that they ask patients about current/previous use of alcohol, while in Moldova and Armenia, this percentage was at 57.0% and 64.2%, respectively.

When asked if respondents had ever detected a suspicious lesion for oral cancer, 50.7% of dentists in Moldova, 82.7% in Belarus and 32.0% in Armenia confirmed that they had. More than two-thirds of dentists in Belarus and Armenia and more than half in Moldova have referred suspected oral cancer patients to a specialist. Less than 30% of the dentists reported that they had taken a biopsy of the oral mucosa (Table 4).

**Table 4:** Dentists’ practices when detecting a suspicious lesion

	Moldova (N = 268)	Belarus (N = 704)	Armenia (N = 328)
Ever detected a suspicious lesion for oral cancer			
Yes	136 (50.7)	582 (82.7)	105 (32.0)
No	111 (41.4)	88 (12.5)	198 (60.4)
Do not remember	20 (7.5)	33 (4.7)	25 (7.6)
Referred to specialist			
Yes	156 (58.2)	526 (74.7)	239 (72.9)
No	93 (34.7)	117 (16.6)	62 (18.9)
Do not remember	18 (6.7)	58 (8.2)	27 (8.2)
Performed biopsy of oral mucosa			
Yes	47 (17.5)	88 (12.5)	98 (29.9)
No	216 (80.6)	607 (86.2)	214 (65.2)
Do not remember	5 (1.9)	9 (1.3)	16 (4.9)

Data are expressed as n (%). Percentages are calculated as n/N

Some figures are subject to missing data; values may not add up to total sample

The vast majority of dentists agreed or strongly agreed that it is the role of the dentist to perform oral mucosal examination (90.6% in Moldova, 98.6% in Belarus, and 70.6% in Armenia). Only 48.4% of dentists in Belarus agreed or strongly agreed that they can influence a patient to reduce/quit smoking or drinking alcohol, while in Moldova and Armenia, this percentage was at 67.2% and 60.4%, respectively.

### Perceived barriers to perform oral mucosal examination

**Fig. 2:**
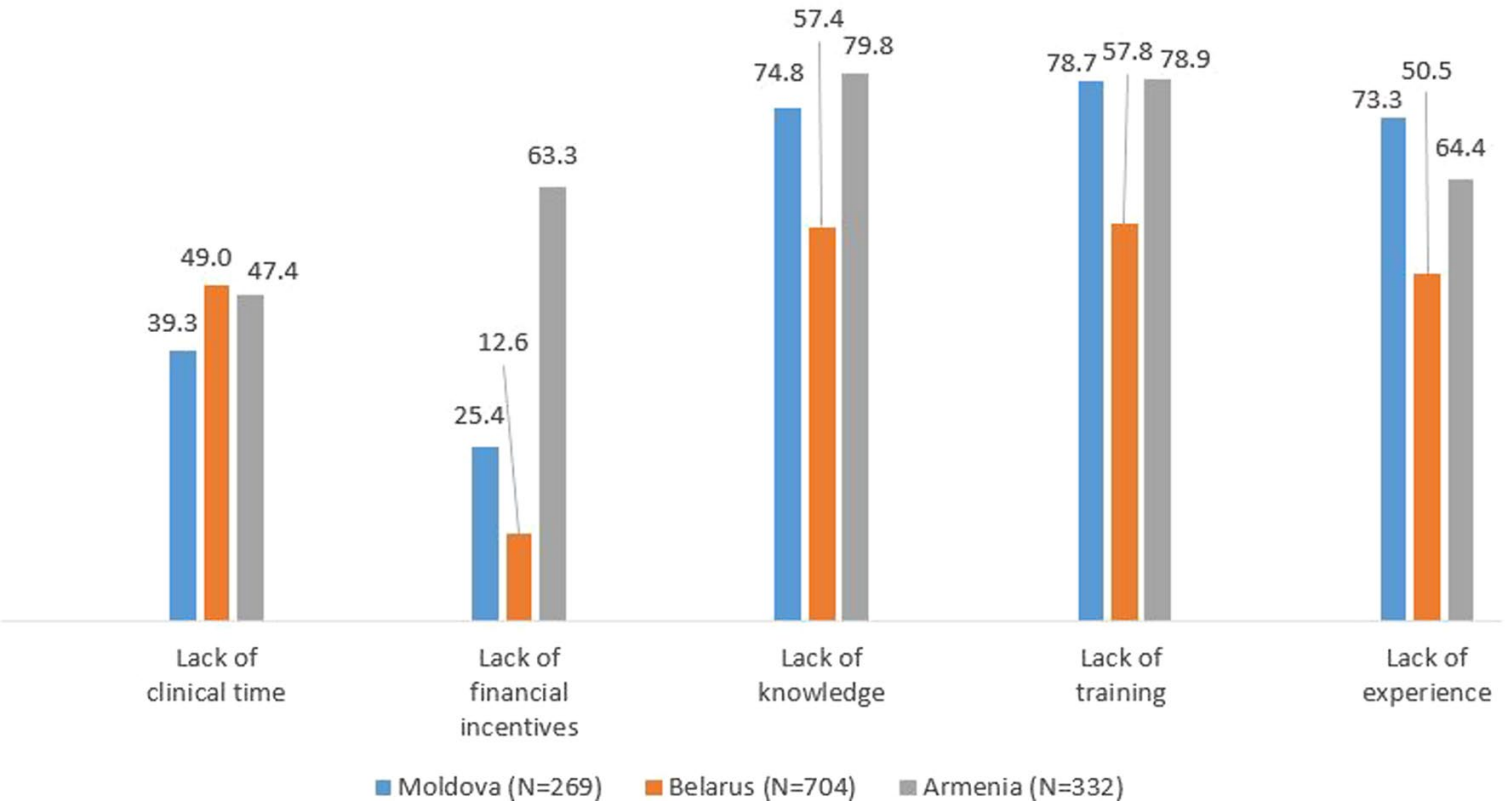
Perceived barriers to perform oral mucosal examination. Data are expressed as percentages

The most commonly listed barriers to perform oral mucosal examination in all three countries were the lack of training, knowledge, and experience (Fig. 2). Moreover, in Armenia, 63.3% of dentists identified the lack of financial incentives as a barrier for performing oral mucosal examination, while in Moldova and Belarus, fewer dentists considered it as a barrier.

## Discussion

As dentists should play a key role in oral cancer prevention and early detection, they need to possess a thorough knowledge about oral cancer, its clinical signs and symptoms, and its risk factors. To the best of our knowledge, this is the first study to assess the knowledge, opinions, and practices of dentists toward oral cancer prevention and oral mucosal examination in Moldova, Belarus, and Armenia.

Overall, participants’ characteristics were comparable across the three countries. However, a striking difference between the three countries was that more than 60% of dentists in Moldova and almost 80% in Armenia were working exclusively in the private sector, while in Belarus, more than 60% were employed in the public sector. Indeed, in Armenia, and especially in Yerevan, the vast majority of dental services and dental equipment support have been privatized [35]. Anecdotal evidence has also suggested that because of low incentives and salaries, many dentists in Armenia have moved from the public to the private sector [35]. Similarly, in Moldova, most oral health care is provided privately [36]. By contrast, in Belarus, state dental clinics are predominant, as private dentistry is not well-developed due to the underdevelopment or lack of insurance systems [37].

The study results showed that more than 83% of respondents were aware of tobacco as a risk factor for oral cancer. By contrast, abusive use of alcohol was less commonly identified as a risk factor. In addition, only 36.5% of dentists in Belarus noted that they ask patients about their current/previous use of alcohol. These findings are concerning, as a strong association between abusive use of alcohol and oral cancer has been noted [38]. This association highlights the importance of dental practitioners’ awareness on abusive alcohol use as a risk factor, particularly in countries with a high alcohol consumption such as Moldova and Belarus [30]. Thus, it is important that dentists in these countries provide adequate information to their patients about the impact of alcohol on oral cancer.

Viral infections with high-risk human papillomavirus (HPV) were also identified as a risk factor for oral cancer by a relatively high percentage of dentists in the present study, although HPV is mainly known as a risk factor for oropharyngeal cancers [39] and not for oral cancers [40]. While HPV has been identified in around 70–90% of oropharyngeal cancer cases, the average prevalence of HPV-positive oral cancer was estimated at 4.4% in a systematic review evaluating the prevalence of HPV-positive OSCC using *E6/E7* mRNA expression analysis [40]. This low prevalence of HPV-positive OSCC challenges the view that HPV is a possible etiological factor in oral cancer. Nevertheless, due to the proximity of the oropharynx to the oral cavity and that the upper part of oropharynx should be examined by dentists, dentists should know about the association between HPV and oropharyngeal cancer [39].

At an early stage, oral cancer is usually asymptomatic and might be difficult to detect due to clinical similarities and the size of the lesion. For this reason, dental practitioners must be conscious about what to look for and where to look in order to detect oral cancer at an early stage [7, 41, 42]. The mobile/anterior 2/3 of the tongue, the lateral surface of the tongue, and floor of the mouth are considered as the most common sites for oral cancer [43]. All three sites were correctly identified as the most common locations for oral cancer by less than 52% of the study population. By contrast, a recent systematic review measuring knowledge, attitudes, and practices regarding OSCC among dental practitioners revealed a good level of knowledge with regard to the common high-risk sites of oral cancer development such as tongue (up to 81%) and floor of the mouth (up to 86%) [9]. Leukoplakia and erythroplakia are the most common oral potentially malignant disorders (OPMDs) [44]. Leukoplakia was correctly identified by most of the dentists in all three countries. The respondents appeared to be less familiar with erythroplakia, so that it was correctly identified by only 30%, 49% and 20% of dentists in Moldova, Belarus and Armenia, respectively. Looking at similar studies, 87% of dentists in Yemen reported leukoplakia and erythroplakia as OPMDs [19]. An interesting point is that in Armenia, aphthous ulceration was incorrectly identified as an OPMD by 40.2% of the dentists. Aphthous ulceration in most cases is a harmless yellowish-white lesion and the outbreak heals within 7–14 days [45].

The most common OPMDs present clinically as small, painless, red or white, as well as ulcerations with coarseness [4]. Small, painless, indurated ulceration was correctly identified as a clinical property of an early oral cancer lesion by 33.9–63.4% of the dentists, and small, painless white or red by less than 35% of dentists in all three countries. In a study from Sudan, 80.5% of dentists correctly identified “white or red areas” as a marker of early oral cancer lesions, and more than 80% correctly reported that oral cancer at an early stage is usually painless [46]. This is in contrast to an Iranian study in which around 30% of dentists considered small, painless and red lesions as an early sign of oral cancer [47]. A range in the level of dentists’ knowledge by country of practice was similarly noted in the present study, which might be related to different educational backgrounds, different training opportunities, and different professional environments.

With regard to oral mucosal examination, most dentists included in this study reported that they provide oral mucosa examination to all new and recall patients. Although these results are encouraging, they warrant caution, as with the questionnaire used in the present study it is not possible to determine the accuracy of oral mucosal examination. Data from the study conducted in Iran revealed that approximately 38% of dentists performing oral mucosa examination did not properly examine the tongue. Only 40% of them inspected the area under the tongue and only 15% examined the rim of the tongue [47].

Our study found that a high percentage of dentists in Belarus, and to a lesser extent in Moldova, have already detected a suspicious lesion for oral cancer. By contrast, most dentists have never performed a biopsy of oral mucosa throughout their career. This suggests, that when detecting a suspicious lesion, dentists tend to refer the patient to an appropriate specialist for an examination and biopsy such as an oral surgeon or an oral pathologist. Indeed, oral cancer is best managed by specialists with advanced training in oral pathology or oral surgery [33]. This practice is in line with the previous study from Iran [47], and with a study from the United Arab Emirates in which only 9.9% of dentists reported feeling comfortable performing a biopsy [48]. As clinical properties of an early cancer lesion are usually more obvious at an advanced stage, biopsy and histopathological examination can help diagnose an early-stage cancer [4, 49, 50]. Among the different dental specialties, pediatric dentists are the least likely to see a suspicious lesion for oral cancer.

Most of the participants commonly listed lack of training, knowledge, and experience as the main barriers to perform oral mucosa examination. In studies from Yemen [19] and Jordan [51], more than 80% of dentists perceived their knowledge about oral cancer as not up to date, which reveals the importance of continuous education on oral cancer. Educational interventions may be beneficial for dentists in all three studied countries and possibly reduce some of the barriers mentioned by the participants of the present study. Perceived barriers for performing oral mucosa examination were very similar between the countries, except for the lack of financial incentives that was largely reported as a barrier in Armenia. Unfortunately, in Armenia, low wages and difficult work conditions have been extensively reported for dentists and other dental health professionals working in both the public and the private sector [35].

As in many other surveys, this study has some limitations, primarily its relatively low response rate (37.2%). However, the response rate in the present study was higher than that reported in previous similar studies (< 30%) [3, 52]. Nevertheless, the response rate might be attributed to the questionnaire length, as it was part of a more comprehensive questionnaire on prevention of oral diseases and consisted of 70 questions in total. This may have been interpreted as cumbersome and could have been a reason for reluctance to participate in the study. The low response rate, especially in Moldova and Armenia, may have led to non-response bias. However, we believe that the extent of differences between responders and non-responders in our study is narrow, given the broad representation of dentists (working in both the private and the public sectors) from different dental specialties [52]. In addition, the lists of dental clinics, which were obtained from the local coordinators, might have been somewhat outdated, hence possibly excluding certain new clinics. Moreover, since the study was based on a self-administered questionnaire, potential biases caused by self-reporting (i.e., recall bias and social desirability bias) cannot be excluded. To minimize social desirability bias, anonymous questionnaires were used in the present study. Furthermore, since participation in this study was voluntary, selection bias related to personal interests of clinicians cannot be ruled out. Lastly, since this study was restricted to the capital cities of Moldova, Belarus and Armenia, it is challenging to generalize these results to other parts of the country as well as to other countries. Nevertheless, our study had several strengths, including its large and multinational sample size, the very few missing data, and its novelty. This study also addresses an important health issue that challenges dentists in these three post-Soviet nations.

## Conclusions

This cross-sectional study conducted among dentists in Moldova, Belarus, and Armenia highlights strengths as well as gaps in dentists’ knowledge and practices related to oral cancer prevention and early detection. Data from the current study can be used as a foundation for future educational programs for dentists and can help reinforce the dental curriculum in order to enhance awareness and knowledge related to oral cancer prevention.

## Supplementary Information

**Additional file 1.** Questionnaire used in this study

## Data Availability

The datasets used for the current study are available from the corresponding author on reasonable request.

## Abbreviations

CI: Confidence interval
HPV: Human papillomavirus
OPMD: Oral potentially malignant disorder
OR: Odds ratio
OSCC: Oral squamous cell carcinoma
SD: Standard deviation
