# Supplementary material for: Knowledge, opinions, and practices related to oral cancer prevention and oral mucosal examination among dentists in Moldova, Belarus and Armenia: a multi-country cross-sectional study
Source: BMC Oral Health. 2021 Dec 18;21:652. doi: 10.1186/s12903-021-02011-2 (PMC8684171; doi:10.1186/s12903-021-02011-2)
Supplement: Supplementary file 1 — Additional file 1. Questionnaire used in this study [file 12903_2021_2011_MOESM1_ESM.docx]

**Additional file 1.** Questionnaire used in this study

**No: ...**

**Personal data**

1. Age

……………………….

1. Gender
   1. Female
   2. Male
2. How long have you been working as a dentist?

........................

1. Where did you complete your basic degree as a dentist?
   1. In Moldova/Armenia/Belarus
   2. Abroad
2. Current job(s)
   1. Dentist in private clinic
   2. Dentist in public clinic/university
   3. Both
3. Type of practice
   1. Solo
   2. Non-solo (several dentists in a practice)
4. Specialty
   1. General dentistry
   2. Pediatric dentistry/orthodontics
   3. Restorative/endodontics
   4. Periodontics
   5. Prosthodontics
   6. Oral surgery
   7. Oral pathology
5. Patient group you presently work with
   1. Children (0-18)
   2. Adults
   3. Mixed

**Oral mucosal screening and oral cancer prevention**

1. Do you check all new patients for oral mucosal (buccal, lip, floor of the mouth, tongue, base of tongue, palate, retromolar area and palatopharyngeal arches) lesions?
   1. Yes
   2. No
   3. Do not remember
2. Do you check all recall patients for oral mucosal lesions?
   1. Yes
   2. No
   3. Do not remember
3. Have you ever detected a suspicious lesion for oral cancer?
   1. Yes
   2. No
   3. Do not remember
4. Have you ever referred further to a specialist for a suspicious lesion for oral cancer?
   1. Yes, if yes, write where (oral surgeon, ear-nose-throat doctor, family doctor?) …………………………….
   2. No
   3. Do not remember
5. Have you ever performed biopsy of oral mucosa?
   1. Yes
   2. No
   3. Do not remember
6. Checking oral mucosa should occur for all new patients.
   1. Strongly disagree
   2. Disagree
   3. Do not know
   4. Agree
   5. Strongly agree
7. Checking oral mucosa should occur for all recall patients.
   1. Strongly disagree
   2. Disagree
   3. Do not know
   4. Agree
   5. Strongly agree
8. Checking oral mucosa should be targeted to those at high risk of developing oral cancer.
   1. Strongly disagree
   2. Disagree
   3. Do not know
   4. Agree
   5. Strongly agree
9. Lack of clinical time is a barrier to checking oral mucosa.
   1. Strongly disagree
   2. Disagree
   3. Do not know
   4. Agree
   5. Strongly agree
10. Lack of financial incentives is a barrier to checking oral mucosa.
    1. Strongly disagree
    2. Disagree
    3. Do not know
    4. Agree
    5. Strongly agree
11. Lack of knowledge in performing oral mucosa examination is a barrier to checking oral mucosa.
    1. Strongly disagree
    2. Disagree
    3. Do not know
    4. Agree
    5. Strongly agree
12. Lack of training in performing oral mucosa examination is a barrier to checking oral mucosa.
    1. Strongly disagree
    2. Disagree
    3. Do not know
    4. Agree
    5. Strongly agree
13. Lack of experience in performing oral mucosa examination is a barrier to checking it.
14. Strongly disagree
15. Disagree
16. Do not know
17. Agree
18. Strongly agree
19. Do you consider use of tobacco as a risk factor for oral cancer?
    1. Yes
    2. No
    3. Do not know
20. Do you consider viral infection with HPV as a risk factor for oral cancer?
    1. Yes
    2. No
    3. Do not know
21. Do you consider abusive use of alcohol as a risk factor for oral cancer?
    1. Yes
    2. No
    3. Do not know
22. Do you consider older age as a risk factor for oral cancer?
    1. Yes
    2. No
    3. Do not know
23. Do you consider low consumption of fruits and vegetables as a risk factor for oral cancer?
    1. Yes
    2. No
    3. Do not know
24. Do you consider prior oral cancer lesion as a risk factor for oral cancer?
    1. Yes
    2. No
    3. Do not know
25. Do you consider the most common sites for oral cancer to be *(Tick those that apply)*
    1. all sites equally
    2. floor of the mouth
    3. buccal/lip mucosa
    4. hard palate
    5. soft palate
    6. retromolar region/palatopharyngeal arches
    7. tongue
    8. rim (sides) of tongue
    9. Do not know
26. The two lesions most likely to be pre-cancerous are (*You should tick 2 answers)*
    1. Morbus Chron
    2. Erythroplakia
    3. Blue nevus
    4. Leukoplakia
    5. Aphtha
    6. Do not know
27. Most common clinical properties of an early cancer lesion *(Tick those that apply)*
    1. small, painless white area
    2. small, painless red area
    3. small, painless, indurated ulceration
    4. small, painful, indurated ulceration
    5. Do not know
28. Do you ask your patients about current/previous use of tobacco?
    1. Yes
    2. No
    3. Do not remember
29. Do you ask your patients about current/previous use of alcohol?
    1. Yes
    2. No
    3. Do not remember
30. Do you ask your patients about family history of cancer?
    1. Yes
    2. No
    3. Do not remember
31. You in current role as a dentist can influence a patient to reduce/quit smoking or drinking alcohol.
    1. Strongly disagree
    2. Disagree
    3. Do not know
    4. Agree
    5. Strongly agree
32. You in current role as a dentist should provide smoking or alcohol cessation advice.
    1. Strongly disagree
    2. Disagree
    3. Do not know
    4. Agree
    5. Strongly agree
33. It is the role of dentists to perform oral mucosal screening.
    1. Strongly disagree
    2. Disagree
    3. Do not know
    4. Agree
    5. Strongly agree
34. It is the role of medical doctors/family doctor/specialists to perform oral mucosal screening.
    1. Strongly disagree
    2. Disagree
    3. Do not know
    4. Agree
    5. Strongly agree
35. Where did you get the information about oral mucosal screening and oral cancer prevention? *(Tick those that apply)*
36. Undergraduate courses
37. Postgraduate courses
38. Textbooks
39. Scientific journals
40. Dental congresses
